# Angle-dependent electron-electron correlation in the single ionization of H_2_ in strong laser fields

**DOI:** 10.1038/s41598-018-33015-8

**Published:** 2018-10-08

**Authors:** Wan-Yang Wu, Feng He

**Affiliations:** 1 Key Laboratory for Laser Plasmas (Ministry of Education) and School of Physics and Astronomy, Shanghai Jiao Tong University, Shanghai, 200240 China; 2 Collaborative innovation center of IFSA (CICIFSA), Shanghai Jiao Tong University, Shanghai, 200240 China

## Abstract

The one-photon ionization and tunneling ionization of H_2_ exposed to strong XUV and infrared laser pulses are studied by numerically simulating the four-dimensional time-dependent Schrödinger equation, which includes two-electron dynamics for arbitrary angle between the molecular axis and the laser polarization direction. In the one-photon single ionization of H_2_, one electron escapes fast and the other bound electron is not disturbed but remains in coherent superposition of two electronic states of ${{\bf{H}}}_{{\bf{2}}}^{{\boldsymbol{+}}}$. In another case, under the irradiation of strong infrared laser pulses, one electron tunnels through the laser-dressed Coulomb barrier, and the other bound electron has enough time to adapt to the potential of ${{\bf{H}}}_{{\bf{2}}}^{{\boldsymbol{+}}}$ and thus is prone to transfer to the ground electronic state of ${{\bf{H}}}_{{\bf{2}}}^{{\boldsymbol{+}}}$. In the intermediate regime, between the one photon and tunneling regimes, this electron-electron correlation depends strongly on the laser frequency, laser intensity and on the angle between laser polarization and the molecular axis.

## Introduction

Ionization lies at the heart of diverse research directions in atomic and molecular physics, such as high harmonic generation^1,2^, photoelectron holography^3^, Auger decay^4^, shake-off^5,6^ and shake-up^7^. The Keldysh parameter^8^ *γ* serves as a guide to understand ionization behaviors in different regimes. The Keldysh parameter is defined as $\gamma =\sqrt{{I}_{p}\mathrm{/2}{U}_{p}}$, where *I*_*p*_ is the ionization potential, and *U*_*p*_ is the averaged quiver energy for a free electron in a laser field. When $\gamma \gg 1$, the laser period is much shorter than the typical time scale for the electron movement, thus, the electron sees a fast oscillator. In this scenario, photoemission has traditionally been studied in the frequency domain, and the photon absorption is assumed to be instantaneous^9,10^. The sudden removal of the photoelectron will cause a change in the potential so that the second electron could be shaken off with a certain probability, resulting an immediate double ionization. Electron correlations in the initial state play a key role in the shake-off mechanism^11^. The interaction between the departing electron and bound electrons also exists after photon absorption, and the second electron may be knocked out by the photoelectron in an (e,2e)-like process^12–14^, which is viewed as a final-state correlation in the knockout mechanism^15^.

On the other hand, when $\gamma \ll 1$, the electron sees a very slow oscillating electric field, giving itself enough time to adapt to the laser-distorted Coulomb potential and tunnel through the Coulomb barrier^16–19^. For a complex atom or molecule, when a valence electron is tunneling ionized, the simultaneous excitation of the parent ion has long been considered negligible. Until recently, simulations^20–22^ and precise measurements^23–26^ suggested that electronic excitations exist in the recollision-free tunneling ionization for large molecules such as CO_2_, H_2_O, HCl, and so on. However, the electron-electron correlation during the tunneling ionization has not yet been studied via *ab initio* calculations.

One-photon ionization of H_2_ has been extensively studied experimentally^27–29^, with specific emphasis on two-electron emission in single photon absorption. In tunneling ionization of H_2_, the first experiment has demonstrated that excitations induced by electron-electron correlation are small^30^. Theoretically, H_2_ serves as a useful prototype for understanding the electron-electron correlation during the ionization process^31–33^. These studies focused on two electron ejection and lacked adequate discussions of electronic excitation in the case that only one electron is ionized. The photoelectron momentum distributions corresponding to single ionization of ${{\rm{H}}}_{2}^{+}$ or H_2_ in XUV fields have been numerically studied^34,35^, which show that the photoelectron emission depends sensitively on the photon energy and internuclear distance. So far, in the case of tunneling ionization, full two-electron dynamic has not been yet investigated due to the immense computation effort involved for solving TDSE in six-dimension, which was alleviated by confining both electrons along the molecular axis in these *ab initio* simulations^36,37^.

In this paper, we studied the electron correlation in single ionization processes of H_2_ by numerically simulating the four-dimensional (4D) time-dependent Schrödinger equation (TDSE), in both the tunneling and one-photon regimes with a linearly polarized light. It involves a complete treatment of electron-electron correlation in the initial and final states, as well as during the ionization process. The angle between the laser polarization direction and the molecular axis may affect the laser-electron coupling, as well as the electron-electron correlation. Our simulation results show that two electrons are strongly correlated in the tunneling ionization process of H_2_. However, in one-photon single ionization, one electron leaves too fast and the other one is unable to make a prompt response, and thus the correlation during the single ionization process is negligible.

## Methods

### TDSE simulations

The 4D TDSE of H_2_ is given by (atomic units are used throughout unless stated otherwise)

1 $$i\frac{\partial }{\partial t}{\rm{\Psi }}({{x}}_{{\rm{1}}},\,{{\rm{y}}}_{{\rm{1}}},\,{{x}}_{{\rm{2}}},\,{{\rm{y}}}_{{\rm{2}}};\,t)=[{H}_{0}(t)+{H}_{int}(t)]\,{\rm{\Psi }}({{x}}_{{\rm{1}}},\,{{\rm{y}}}_{{\rm{1}}},\,{{x}}_{{\rm{2}}},\,{{\rm{y}}}_{{\rm{2}}};\,t),$$

in which the field-free Hamiltonian *H*_0_ is

2 $${H}_{0}=\frac{{p}_{{x}_{1}}^{2}}{2}+\frac{{p}_{{y}_{1}}^{2}}{2}+\frac{{p}_{{x}_{2}}^{2}}{2}+\frac{{p}_{{y}_{2}}^{2}}{2}+V({x}_{1},\,{y}_{1},\,{x}_{2},\,{y}_{2}),$$

with *p* the momentum operator for both electrons and the Coulomb potential

3 $$V({x}_{1},\,{y}_{1},\,{x}_{2},\,{y}_{2})=\frac{1}{\sqrt{{({x}_{1}-{x}_{2})}^{2}+{({y}_{1}-{y}_{2})}^{2}+\alpha }}-\sum _{s=\pm 1}\sum _{i\mathrm{=1}}^{2}\frac{1}{\sqrt{{({x}_{i}+sR\mathrm{/2)}}^{2}+{y}_{i}^{2}+\beta }}+\frac{1}{R}\mathrm{.}$$

Here, *α* and *β* are the soft-core parameters, and *R* is the internuclear distance. The laser-molecule coupling is expressed in velocity gauge by

4 $${H}_{int}(t)=({p}_{{x}_{1}}{\hat{e}}_{x}+{p}_{{y}_{1}}{\hat{e}}_{y}+{p}_{{x}_{2}}{\hat{e}}_{x}+{p}_{{y}_{2}}{\hat{e}}_{y})\cdot {\bf{A}}(t),$$

where ${\bf{A}}(t)=-{\int }_{-\infty }^{t}{\bf{E}}(t^{\prime} )dt^{\prime}$ is the laser vector potential, and the linearly polarized **E**(*t*) is expressed as

5 $${\bf{E}}(t)={E}_{0}\,f(t){\rm{s}}{\rm{i}}{\rm{n}}(\omega t)({\hat{e}}_{x}{\rm{c}}{\rm{o}}{\rm{s}}{\boldsymbol{\theta }}+{\hat{e}}_{y}{\rm{s}}{\rm{i}}{\rm{n}}{\boldsymbol{\theta }})\mathrm{.}$$

Here *θ* is the angle between the molecular axis and the laser polarization direction, *ω* is the laser angular frequency, and the pulse envelope *f*(*t*) = sin^2^(*πt*/*τ*) for 0 ≤ *t* ≤ *τ* and zero elsewhere. The amplitude ${E}_{0}=\sqrt{({I}_{0}\mathrm{/3.51}\times {10}^{16})}$. For simulations in this paper, the laser field intensity *I*_0_ is 10^14^ W/cm^2^ except otherwise stated, and the pulse duration *τ* = 5 T and 0.5 T for one-photon and tunneling ionization, respectively. *T* = 2*π*/*ω* is the laser period.

We obtained the ground state Ψ_0_(*x*_1_, *y*_1_, *x*_2_, *y*_2_) of Eq. 1 by imaginary time propagation^38^. The Crank-Nicholson method^39^ was used for the wave function propagation with spatial grids Δ*x*_1_ = Δ*y*_1_ = Δ*x*_2_ = Δ*y*_2_ = 0.3 a.u. and the time step Δ*t* = 0.05 a.u. We tested the convergence of the above time and distance steps. The 4D simulation box *x*_1_ − *y*_1_ − *x*_2_ − *y*_2_ is sampled by the grids 400^4^ or 800^4^ when XUV or infrared laser pulses are used, respectively. The simulation box is big enough to contain all single ionization events except for the two-XUV-photon single ionization with much smaller probability, which is absorbed by the mask function $co{s}^{\mathrm{1/6}}$ at the boundaries. In this 4D TDSE model, we set *α* = 1.0 and *β* = 0.35^35^. Wave packets within the area $(\sqrt{{x}_{1}^{2}+{y}_{1}^{2}}-{R}_{c})\times (\sqrt{{x}_{2}^{2}+{y}_{2}^{2}}-{R}_{c}) < 0$ are regarded as the single ionization, where *R*_*c*_ = 20 a.u. Note that we have varied *R*_*c*_ around 20 a.u. and there is no visible difference for observations. The Fourier transform of the ionized wave packet gives the photoelectron momentum distribution. After the end of laser field, we kept propagating the wave function for extra time until the momentum distributions had already converged. Concerning the opposite spins for the two electrons, the whole spatial wave packet of H_2_ satisfies the two-electron exchange symmetry. For expressing clearly and simply, in the following discussions we optionally chose the ionized electron with coordinates (*x*_2_, *y*_2_) and the bound electron with coordinates (*x*_1_, *y*_1_). To be more accurately, one should treat two electrons completely equally. However, our treatment does not change any physical conclusions.

To identify which electronic state is for ${{\rm{H}}}_{2}^{+}$, we projected the singly ionized molecular wave packet of H_2_ onto the field free electronic eigenstates ***ψ***_*j*_(*x*_1_, *y*_1_) of ${{\rm{H}}}_{2}^{+}$, which were obtained by numerically solving Eq. 1 where terms related to *x*_2_ and *y*_2_ had been omitted. The *j* in ***ψ***_*j*_(*x*_1_, *y*_1_) labels the *j*-th electronic state of ${{\rm{H}}}_{2}^{+}$.

### Strong field approximation

To verify how significant is the electron-electron correlation during the photoionization and to make a contrast with the TDSE results, we used the strong field approximation (SFA) to calculate the photoelectron momentum distribution of the single ionization of H_2_. The transition amplitude is written as^40^

6 $${M}_{j}({{\bf{p}}}_{2})=-\,i{\int }_{0}^{\tau }\,\langle {\varphi }_{{\bf{p}}}(t)|{\bf{A}}(t)\cdot {{\bf{p}}}_{2}|{{\boldsymbol{\Psi }}}_{0}({{x}}_{{\rm{1}}},\,{{y}}_{{\rm{1}}},\,{{x}}_{{\rm{2}}},{{y}}_{{\rm{2}}})\rangle dt,$$

where the initial state **Ψ**_0_(*x*_1_, *y*_1_, *x*_2_, *y*_2_) is the ground state of H_2_ obtained by numerically solving Eq. 1 in the field free case. ${\varphi }_{p}(t)={{\boldsymbol{\psi }}}_{j}({x}_{1},\,{y}_{1})\,\exp \,(i{p}_{{x}_{2}}{x}_{2}+i{p}_{{y}_{2}}{y}_{2})\,\exp \,[\,-\,iS(t)-It]$ is the singly ionized final state with the ion ${{\rm{H}}}_{2}^{+}$ in the *j*-th electronic state and a freed electron with momentum ${{\bf{p}}}_{2}=({p}_{{x}_{2}},{p}_{{y}_{2}})$, where *S* is the Volkov phase, and *I* is the *R*-dependent molecular ionization potential.

Eq. 6 can be rewritten as^41^

7 $$\begin{array}{ll}{M}_{j}({{\bf{p}}}_{2}) & =-i\,\langle {{\boldsymbol{\psi }}}_{j}({x}_{1},{y}_{1})|{{\rm{\Psi }}}_{0}({x}_{1},\,{y}_{1},\,{p}_{{x}_{2}},\,{p}_{{y}_{2}})\rangle F({p}_{{x}_{2}},\,{p}_{{y}_{2}})\\  & =-i{\phi }_{j}({p}_{{x}_{2}},\,{p}_{{y}_{2}})F({p}_{{x}_{2}},\,{p}_{{y}_{2}}),\end{array}$$

with the laser action

8 $$F({p}_{{x}_{2}},\,{p}_{{y}_{2}})={\int }_{0}^{\tau }\,dt\,\exp \,[i(S(t)+It)]\,[A(t){p}_{{x}_{2}}{\rm{c}}{\rm{o}}{\rm{s}}\theta +A(t){p}_{{y}_{2}}{\rm{s}}{\rm{i}}{\rm{n}}\theta ]\mathrm{.}$$

The electron-correlation is fully described in time in the case of TDSE. However, the SFA does not include electron-electron correlation as a function of time. By comparing the SFA and TDSE simulation results, one may tell the importance of the electron correlation during the ionization.

## Results and Discussions

### Components of the H_2_ ground state

The ground state of H_2_ can also be rephrased as the correlated wave packets of the ion ${{\rm{H}}}_{2}^{+}$ and the electron, i.e., ${{\rm{\Psi }}}_{0}({x}_{1},\,{y}_{1},\,{x}_{2},\,{y}_{2})={\sum }_{j}\,{\psi }_{j}({x}_{1},\,{y}_{1}){\phi }_{j}({x}_{2},\,{y}_{2})$, where *ψ* and *φ* indicate ${{\rm{H}}}_{2}^{+}$ and the valence electron, respectively. Inversely, by projecting Ψ_0_(*x*_1_, *y*_1_, *x*_2_, *y*_2_) onto different eigenstates of ${{\rm{H}}}_{2}^{+}$, we obtained the electron wave packet associated with different eigenstates of ${{\rm{H}}}_{2}^{+}$, i.e.,

9 $${\phi }_{j}({x}_{2},\,{y}_{2})=\langle {\psi }_{j}({x}_{1},\,{y}_{1}){|{\rm{\Psi }}}_{0}({x}_{1},\,{y}_{1},\,{x}_{2},\,{y}_{2})\rangle \mathrm{.}$$

**Figure 1:**
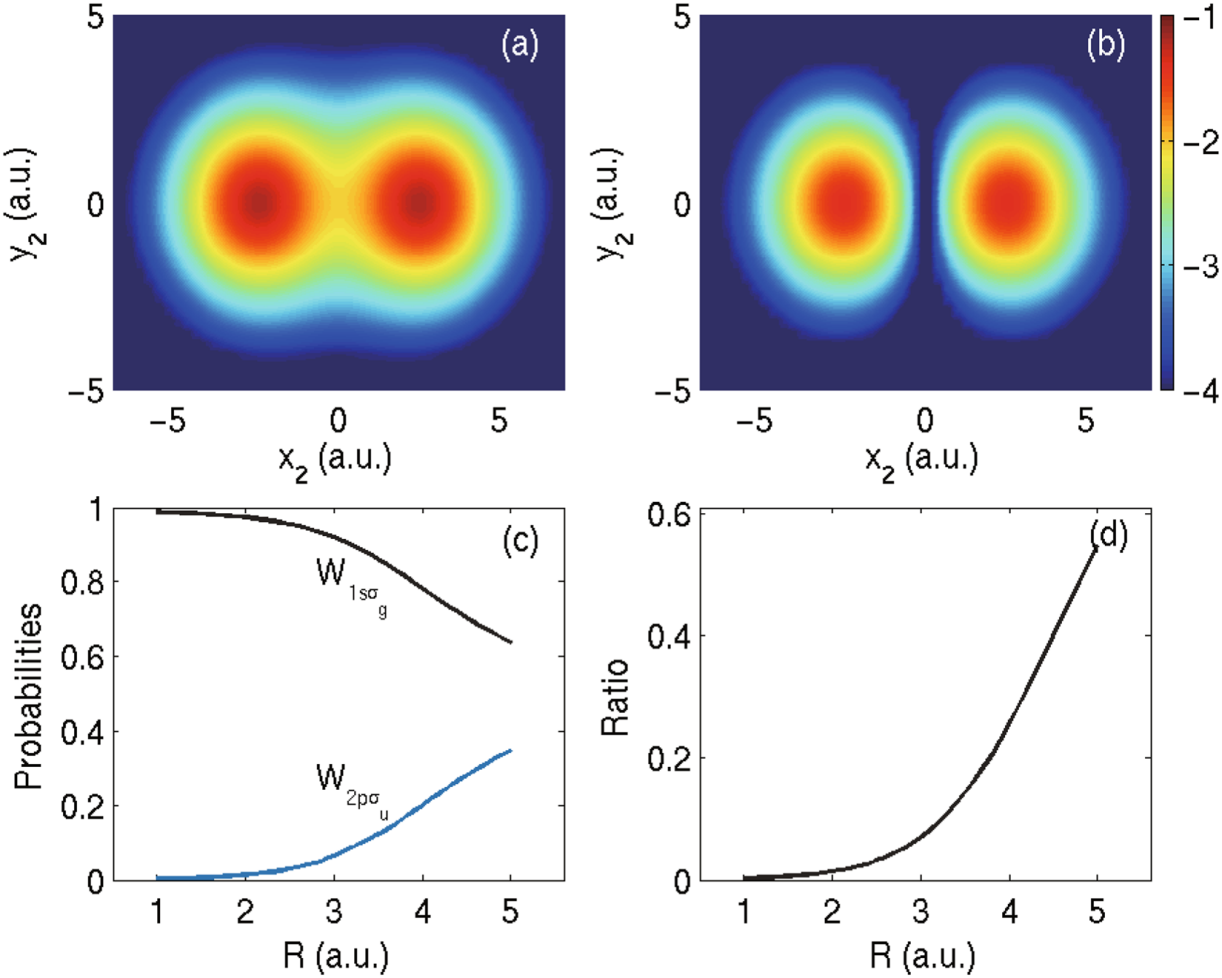
Electron probability distributions obtained by projecting the four-dimensional wave function of H_2_ onto two-dimensional wave function of ${{\rm{H}}}_{2}^{+}$ in (**a**) 1*sσ*_*g*_ state and (**b**) 2*pσ*_*u*_ state, according to Eq. 9. The internuclear distance *R* is 5 a.u. (**c**) Probabilities of ${W}_{1s{\sigma }_{g}}$, ${W}_{2p{\sigma }_{u}}$, and (**d**) The ratio of ${W}_{2p{\sigma }_{u}}/{W}_{1s{\sigma }_{g}}$ as a function of the internuclear distance.

By looking into the proportion of different electronic states of ${{\rm{H}}}_{2}^{+}$ before and after the single ionization of H_2_, one may elucidate the role of the electron-electron correlation during the single ionization process. Figure 1(a,b) show the electron wave function distributions |*ϕ*_*j*_(*x*_2_, *y*_2_)|^2^ with the internuclear distance *R* = 5 a.u. when the associated *ψ*_*j*_(*x*_1_, *y*_1_) of ${{\rm{H}}}_{2}^{+}$ is in the ground or first excited state, respectively. Figure 1(c) shows the *R*-dependent ${W}_{1s{\sigma }_{g}}$ and ${W}_{2p{\sigma }_{u}}$, where ${W}_{1s{\sigma }_{g}}$ and ${W}_{2p{\sigma }_{u}}$ refer to the probabilities of ${{\rm{H}}}_{2}^{+}$ in the ground and first electronic excited states, respectively. The corresponding branching ratio of ${W}_{2p{\sigma }_{u}}/{W}_{1s{\sigma }_{g}}$ is presented in Fig. 1(d). The deviation of the ratio from zero means that it is not accurate to assume H_2_ as the product of the frozen ${{\rm{H}}}_{2}^{+}$ and the valence electron, especially in a large internuclear distance. When the internuclear distance is large, two electrons repel each other and each electron well locates on each nucleus, in other words, the electron is on the superimposed 1*sσ*_*g*_ and 2*pσ*_*u*_ states with similar probabilities. It was analyzed in the pioneer work^30^ that shakeup becomes significant at large internuclear distance due to smaller energy spacings between different states. Here we have shown that the proportion of excited electronic state in the initial H_2_ state increases with the increasing of internuclear distance *R*. The sum of both probabilities shown in Fig. 1(c) is very close to 1, implying that higher excited states of ${{\rm{H}}}_{2}^{+}$ are negligible.

### State of ${{\bf{H}}}_{{\bf{2}}}^{{\boldsymbol{+}}}$ after one-photon ionization

**Figure 2:**
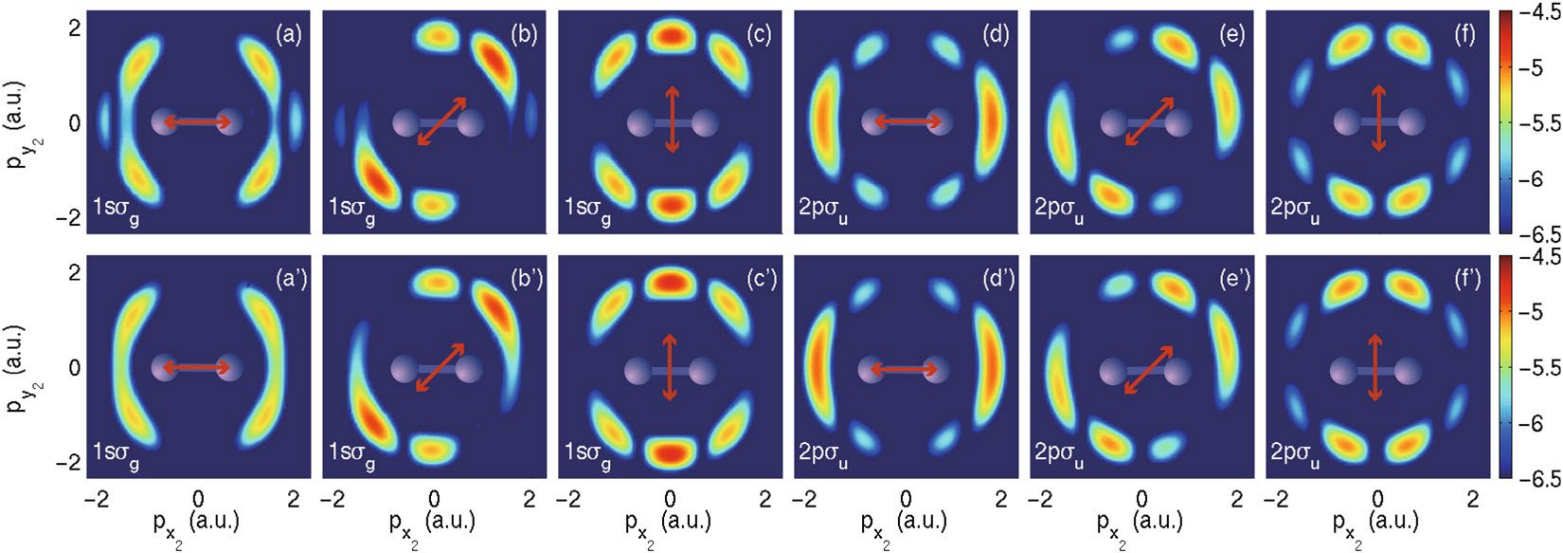
Singly ionized photoelectron momentum distributions at different laser polarization directions of TDSE results governed by Eq. 9 in the top row and SFA results governed by Eq. 6 in the bottom row. (**a**,**c**) For ${{\rm{H}}}_{2}^{+}$ in the 1*sσ*_*g*_ state after single ionization. (**d**,**e** and **f**) For ${{\rm{H}}}_{2}^{+}$ in the 2*pσ*_*u*_ state. The lower row represents corresponding results of SFA. All the calculations were carried out with *ω* = 2.28 a.u. and *R* = 5 a.u.

If one electron in H_2_ is ionized by XUV laser fields, though the remained electron as well as the nuclei seems frozen during the ionization process, the produced ${{\rm{H}}}_{2}^{+}$ is automatically in the superimposed electronic states. Consequently, by projecting the produced ionized wave packets to different electronic eigenstates, we may extract the photoelectron momentum distribution associated with the ${{\rm{H}}}_{2}^{+}$ in a selective electronic state. We numerically decomposed the ionized wave packets by following Eq. 9 in which Ψ_0_(*x*_1_, *y*_1_, *x*_2_, *y*_2_) is replaced by the singly ionized electron wave packet Ψ(*x*_1_, *y*_1_, *x*_2_, *y*_2_, *t*) with $\sqrt{{x}_{1}^{2}+{y}_{1}^{2}} < {R}_{c}$ and $\sqrt{{x}_{2}^{2}+{y}_{2}^{2}} > {R}_{c}$. We used a laser pulse with frequency *ω* = 2.28 a.u. to ionize the ground state H_2_ at the internuclear distance *R* = 5 a.u. The photoelectron momentum distributions associated with ${{\rm{H}}}_{2}^{+}$ in the ground state are shown in Fig. 2(a,b and c) for the angles *θ* = 0, *π*/4 and *π*/2, respectively. The patterns shown in Fig. 2(d,e and f) are the photoelectron momentum distributions associated with ${{\rm{H}}}_{2}^{+}$ in the first excited state. The lower row presents the similar results calculated by the SFA. The stripes of the initial momentum spectrum, which are separated by 2*π*/*R*, leading to the nodes in the photoelectron momentum spectrum in Fig. 2. When the internuclear distance is larger, the separation between neighboring stripes is smaller. Therefore, the laser action will cover more stripes, resulting more nodes in photoelectron momentum distributions. Clearly, the ionized electron wave packets associated with the ground and first excited ${{\rm{H}}}_{2}^{+}$ have distinct photoelectron momentum distributions in Fig. 2. For example at *θ* = *π*/2, the photoelectron associated with ground ${{\rm{H}}}_{2}^{+}$ state has the maximum density at ${p}_{{x}_{2}}=0$ in Fig. 2(c). However, the photoelectron associated with the first excited ${{\rm{H}}}_{2}^{+}$ state has the minimum density at ${p}_{{x}_{2}}=0$ in Fig. 2(f). The photoelectron momentum distributions of these two channels associated with 1*sσ*_*g*_ or 2*pσ*_*u*_ states are distinct, indicating that one can obtain the states of bound electrons by identifying different photoelectron momentum spectra. The complementary maximum and minimum fundamentally depend on the parity of the product of the ionized electron and the associated ${{\rm{H}}}_{2}^{+}$. Both TDSE and SFA simulations start from the same initial state of H_2_ and almost give same results. In the SFA calculations, the two-electron correlation is not included during the ionization process. Thus, the similarity between the two rows in Fig. 2 indicates that the two-electron correlation, which is automatically included in the TDSE simulations, plays negligible roles during the one-photon single ionization.

**Figure 3:**
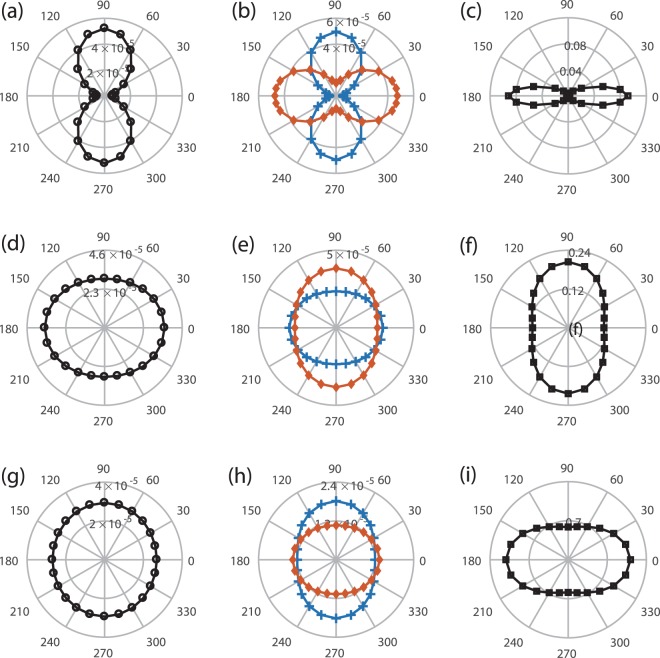
The *θ*-dependent single ionization probability (left column), ionization probabilities associated with the ground ionic state ${W}_{1s{\sigma }_{g}}$ (middle column, crosses) and the first excited ionic state ${W}_{2p{\sigma }_{u}}$ (middle column, diamonds), and the ratio of ${W}_{2p{\sigma }_{u}}/{W}_{1s{\sigma }_{g}}$ (right column). The three rows from up to bottom are for the internuclear distances *R* = 1.67, 3.5 and 5 a.u., respectively. For better clarity, The probabilities of ${W}_{2p{\sigma }_{u}}$ in (**b**) and (**e**) have been multiplied by 100 and 8, respectively. All the calculations were carried out with photon energy *ω* = 2.28 a.u.

Figure 2 has already demonstrated the *θ*-dependence of ionization at *R* = 5 a.u. In Fig. 3, we showed the *θ*-dependent probabilities systematically. The left column of Fig. 3 shows the *θ*-dependent single ionization probabilities for the internuclear distance *R* = 1.67 (a), 3.5 (d) and 5 (g) a.u. The *θ*-dependence of one-photon ionization of H_2_ can be either pronounced or negligible, which strongly relies on the internuclear distance. When the internuclear distance is 1.67 a.u., a spindle profile of the *θ*-dependent ionization probability is obtained. However, an elliptical profile is obtained when the internuclear distances is 3.5 or 5 a.u. The overlap of the laser action and the initial momentum distribution changes with angles *θ* and internuclear distances *R*. Thus, the *θ*- and *R*-dependent ionization probabilities in Fig. 3 can all be explained based on Eq. 7. One may also derive that different laser frequencies will result in different *θ*-dependent ionization probabilities. If the internuclear distance is so large that H_2_ can be regarded as two individual hydrogen atoms, one may expect that neither the tunneling ionization nor the multiphoton ionization will depend on the angle *θ*.

We show in Fig. 3 the probabilities ${W}_{1s{\sigma }_{g}}$ and ${W}_{2p{\sigma }_{u}}$ obtained by projecting the total wave function on the eigenstates 1*sσ*_*g*_ and 2*pσ*_*u*_ of ${{\rm{H}}}_{2}^{+}$. To show more clearly, probabilities of ${W}_{2p{\sigma }_{u}}$ in Fig. 3(b and e) have been multiplied by 100 and 8, respectively. This *θ*-dependence of ${W}_{1s{\sigma }_{g}}$ and ${W}_{2p{\sigma }_{u}}$ can also be explained based on the overlap of the laser action and the electron initial momentum distributions associated with ${{\rm{H}}}_{2}^{+}$ in 1*sσ*_*g*_ and 2*pσ*_*u*_ states. The branching ratio ${W}_{2p{\sigma }_{u}}/{W}_{1s{\sigma }_{g}}$ is shown in the right column in Fig. 3. Due to *R*-dependent correlated initial states as we shown in Fig. 1, *θ*-dependent ratios have distinct shapes at different internuclear distances. The *θ*-dependence of the branching ratios shows that the angle average is necessary if the molecule is not prealigned in laser-molecule interactions.

**Figure 4:**
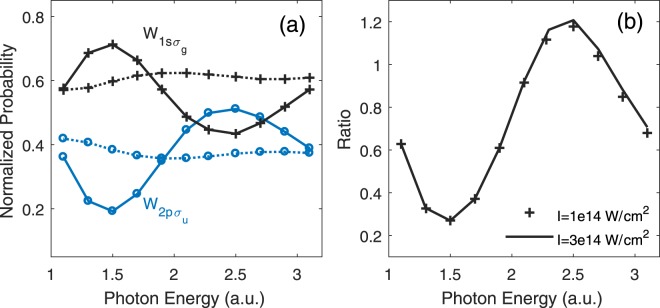
(**a**) The final probabilities of the first two states as a function of the photon energy. The solid and dashed lines represent excitations at polarization angle *θ* = 0 and *π*/2, respectively. The laser intensity *I*_0_ is 10^14^ W/cm^2^. (**b**) Ratios with crosses were obtained by probabilities of solid blue and solid black lines in (**a**), and the black solid line represents ratios of laser field intensity at 3 × 10^14^ W/cm^2^. All the calculations were carried out with internuclear distance *R* = 5 a.u.

Besides the 1*sσ*_*g*_ and 2*pσ*_*u*_ states, some other higher electronic states, though have much smaller populations, also have contributions in the new born ${{\rm{H}}}_{2}^{+}$. For example, at *I*_0_ = 3×10^14^ W/cm^2^, *ω* = 1.9 a.u., and the laser polarization direction is parallel to the molecular axis, the four electronic states of ${{\rm{H}}}_{2}^{+}$, i.e., 1*sσ*_*g*_, 2*pσ*_*u*_, 2*sσ*_*u*_, and 2*pπ*_*u*_ have ratios 0.593: 0.362: 0.037: 0.007. One may expect that relative probabilities for these electronic states also depend on the laser frequency if the internuclear distance is fixed. For photons with other energies, relative probabilities of higher states are still small. Since the two lowest electronic states are dominant in ${{\rm{H}}}_{2}^{+}$, Fig. 4(a) only show the final probabilities of these two states as a function of photon energies. Here, the probabilities of two channels associated with 1*sσ*_*g*_ and 2*pσ*_*u*_ ionic states were normalized to the total single ionization probability induced by each photon energy. The solid and dashed lines represent the laser-molecule interaction geometries at angles *θ* = 0 and *π*/2, respectively. Compared to probabilities ${W}_{1s{\sigma }_{g}}=0.637$ and ${W}_{2p{\sigma }_{u}}=0.348$, shown in Fig. 1(c) at *R* = 5 a.u., we observed that probabilities of the two ionization channels induced by XUV laser fields oscillate around the value obtained by the ground state H_2_. At *θ* = *π*/2, the probability variations of these two states are much smaller. The minima of molecular initial momentum distribution locate along the molecular axis, causing more drastic oscillation for parallel polarization with *θ* = 0. According to Eq. 7, the transition amplitude, and thus the ionization rate, depends on the overlap of $F({p}_{{x}_{2}},\,{p}_{{y}_{2}})$ and the initial wave function in momentum representation ${\phi }_{j}({p}_{{x}_{2}},{p}_{{y}_{2}})$. For the two cases of *ω* = 1.5 and 2.5 a.u., $F({p}_{{x}_{2}},{p}_{{y}_{2}})$ happens to overlap with the minimum and maximum distributions of ${\phi }_{j}({p}_{{x}_{2}},\,{p}_{{y}_{2}})$, which explains the *ω*-dependent ratio shown in Fig. 4(b). The ratio ${W}_{2p{\sigma }_{u}}/{W}_{1s{\sigma }_{g}}$ as a function of photon energy at *θ* = 0, which is shown in Fig. 4(b), does not depend on the laser intensity.

Since the single ionization of H_2_ produces the superimposed electronic eigenstates of ${{\rm{H}}}_{2}^{+}$, a direct outcome is that the remained electron in ${{\rm{H}}}_{2}^{+}$ will hop between two nuclei with a period 2*π*/Δ*E*^42^, where Δ*E* is the energy difference between different electronic states. One may conceive an experiment where the internuclear distance of the neutral H_2_ reaches the critical value for charge-resonance enhanced ionization, the single ionization of H_2_ leaves ${{\rm{H}}}_{2}^{+}$ in the superimposed 1*sσ*_*g*_ and 2*pσ*_*u*_ states. The ultrafast hopping could be observed by performing XUV-pump-XUV-probe experiments. Note that in real molecules, the nuclear movement will somehow destroy the electron hopping and the increasing inter-atomic barrier will ultimately freeze the electron localization after the dissociation of ${{\rm{H}}}_{2}^{+}$^43,44^. For the single ionization associated with ${{\rm{H}}}_{2}^{+}$ in the 1*sσ*_*g*_ state, energies of some nuclear states are above the dissociation limit and thus auto dissociation occurs. For the single ionization associated with ${{\rm{H}}}_{2}^{+}$ in the 2*pσ*_*u*_ state, the dissociation with very high kinetic energy release can be observed^45^.

### State of ${{\bf{H}}}_{{\bf{2}}}^{{\boldsymbol{+}}}$ after tunneling ionization

**Figure 5:**
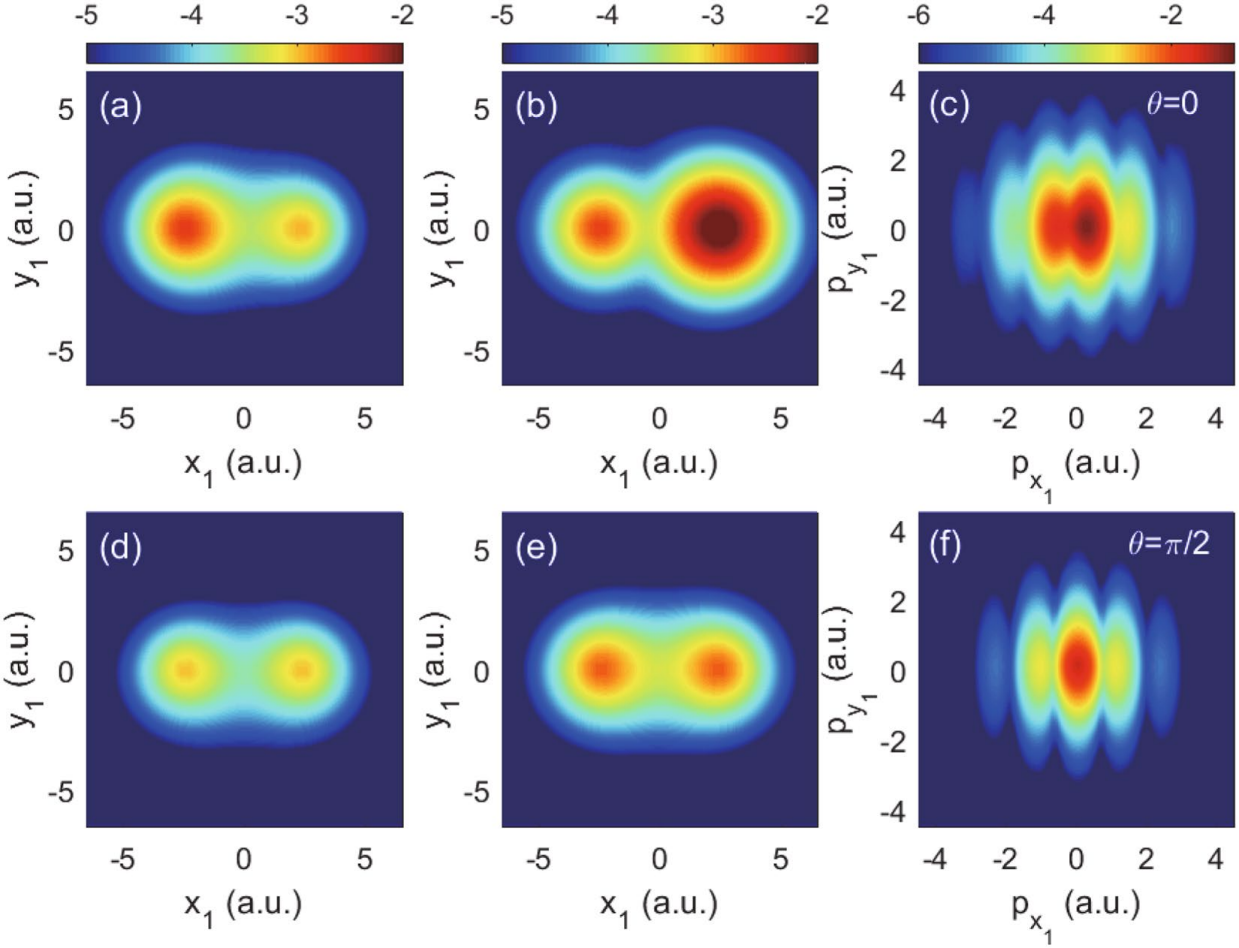
Probability distributions of the bound electron in space (the left and middle columns) and momentum (the right column) representations at polarization angles *θ* = 0 (upper panels) and *θ* = *π*/2 (lower panels). (**a** and **d**) For wave function at *t* = 50 a.u., (**b** and **e**) for wave function at *t* = 85 a.u. (**c** and **f**) are the momentum distributions of (**b** and **e**), respectively. The photon energy is 0.057 a.u. and *I*_0_ = 2 × 10^14^ W/cm^2^. The calculations were carried out with internuclear distance *R* = 5 a.u.

We now study the ionization of H_2_ in a low frequency field, in which case the tunneling picture is adopted to understand the ionization process. To trigger tunneling ionization of H_2_, we used a pulse with half-cycle duration and solved TDSE in length gauge. Because we wanted to investigate recollision-free electron correlations in the linearly polarized electric field, we used such a short pulse to avoid rescattering processes. Moreover, the 4D TDSE in a multi-cycle infrared pulse is still overloaded for supercomputers. Figure 5 shows the bound electron wave function distributions in spatial and momentum representations after the single ionization of H_2_ with *R* = 5 a.u. Figure 5(a,b) present the bound electron distributions in space at t = 50 a.u. and 85 a.u., respectively, from which one can clearly see that this bound electron is hopping between two nuclei of ${{\rm{H}}}_{2}^{+}$. Here, the laser-molecule angle *θ* is 0. Such a strong time-dependent asymmetry is a clear evidence that the electron is in the superimposed states of 1*sσ*_*g*_ and 2*pσ*_*u*_. The simulation shows that branching ratio ${W}_{2p{\sigma }_{u}}/{W}_{1s{\sigma }_{g}}$ at the given laser parameters is 1.2. Figure 5(c) shows the momentum distribution of the bound electron at t = 85 a.u. Due to the superposition of 1*sσ*_*g*_ and 2*pσ*_*u*_ states, the electron momentum distribution is also asymmetric with respect to ${p}_{{x}_{1}}=0$. The lower row of Fig. 5 is the same with the upper row except for the angle *θ* = *π*/2. Different from the case of *θ* = 0, the calculated branching ratio for the bound electron ${W}_{2p{\sigma }_{u}}/{W}_{1s{\sigma }_{g}}$ = 0.05, and thus the time dependent wave function distributions of the remained bound electron shown as Fig. 5(d,e), as well as the momentum distribution in Fig. 5(f) are almost symmetric.

The distinct response of the bound electron in ${{\rm{H}}}_{2}^{+}$ shown in the two rows in Fig. 5 can be explained as following. For *θ* = *π*/2, one electron tunnels through the laser-dressed Coulomb barrier in a more adiabatic way compared to the case of one-photon ionization. The other electron, which is mainly in the superimposed 1*sσ*_*g*_ and 2*pσ*_*u*_ states, has enough time to adapt to the new potential of ${{\rm{H}}}_{2}^{+}$, and therefore transits adiabatically to the ground state of ${{\rm{H}}}_{2}^{+}$ during the single ionization process. In the case of *θ* = 0, during the single ionization of H_2_, the bound electron in ${{\rm{H}}}_{2}^{+}$ is prone to stay in the ground state of ${{\rm{H}}}_{2}^{+}$, at the same time it undergoes a strong laser-induced coupling between 1*sσ*_*g*_ and 2*pσ*_*u*_ states, which is absent in the case of *θ* = *π*/2. Therefore, in the case of *θ* = 0, there are two factors that determine the status of the bound electron in ${{\rm{H}}}_{2}^{+}$ after the single ionization of H_2_, i.e., the laser-induced coupling between 1*sσ*_*g*_ and 2*pσ*_*u*_ states and the adiabatic adaption of the potential of ${{\rm{H}}}_{2}^{+}$. With the increasing of the laser intensity, the transition between 1*sσ*_*g*_ or 2*pσ*_*u*_ states is larger, which leads to a smaller population in the 1*sσ*_*g*_ state. On the other hand, *γ* decreases with the increasing of the laser intensity, and thus the single ionization of H_2_ is more adiabatic, which leads to a larger population in the 1*sσ*_*g*_ state. The competition of these two factors governs the branching ratio ${W}_{2p{\sigma }_{u}}/{W}_{1s{\sigma }_{g}}$.

**Figure 6:**
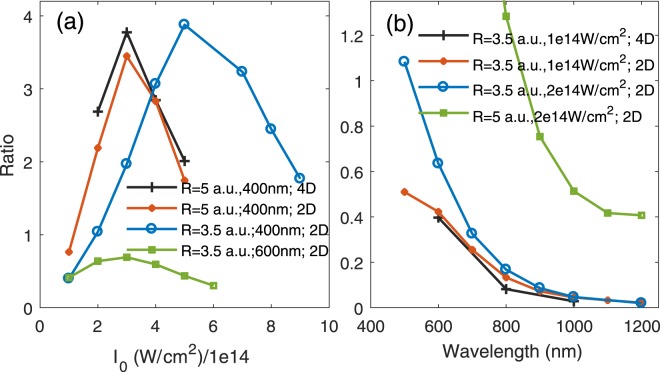
(**a**) Branching ratios as a function of laser field intensity. The black line represents results with 4D H_2_ model at *R* = 5 a.u. and *ω* = 0.114 a.u., the red line is the same with black line except that each electron has only one dimension (2D TDSE). The blue and green lines were obtained by the 2D model for *R* = 3.5 a.u., at *ω* = 0.114 and 0.076 a.u., respectively. (**b**) Branching ratios as a function of laser field wavelength. Other parameters are labeled.

We calculated the branching ratios ${W}_{2p{\sigma }_{u}}/{W}_{1s{\sigma }_{g}}$ as a function of laser intensity at *θ* = 0. The ratio increases with the laser field intensity before reaching a maximum, and then decreases, shown as the black solid line in Fig. 6(a). Along with the 4D TDSE model for H_2_, we also presented the results using the reduced 2D numerical model by confining both-electron movement along the molecular axis. The 2D and 4D calculations show similar profiles. Branching ratios as a function of laser wavelength were also calculated, as shown in Fig. 6(b). At *R* = 3.5 and 5 a.u., differences in energy levels between these two states are 0.17 and 0.06, respectively. For photon energies away from resonant absorption, the laser induced transition between 1*sσ*_*g*_ and 2*pσ*_*u*_ states becomes smaller with the increasing of wavelength. Meanwhile, the single ionization of H_2_ is more close to the adiabatic limit at $\gamma \ll 1$^46,47^. Thus, the branching ratio in Fig. 6(b) decreases monotonically. This monotonically decreasing can also be understood based on the electron-electron correlation. In this tunneling ionization regime, one electron leaves the nuclei so slowly that the two electrons have enough time to correlate each other. The leaving electron adiabatically changes the Coulomb potential exerting on the bound electron, and thus the bound electron adiabatically transits to the ground state of ${{\rm{H}}}_{2}^{+}$.

## Conclusion

In summary, we studied the ionization of H_2_ in either an XUV field or a low-frequency electric field. The 4D model allows us to analyze the photoelectron momentum distribution when the linearly polarized laser pulse crosses the molecular axis with arbitrary angle. Two electrons correlate strongly in H_2_ and thus the initial electronic state of H_2_ can be expanded as superimposed electronic states of ${{\rm{H}}}_{2}^{+}$. During the single-photon ionization of H_2_, the two-electron correlation is negligible and thus the SFA can describe the single ionization of H_2_ reasonably well. In this case, the photoelectron momentum distribution can be regarded as the overlap of the laser action and the electron initial momentum distribution. In the tunneling ionization, especially when the laser polarization direction is perpendicular to the molecular axis, one electron tunnels through the laser-dressed Coulomb potential and the other electron has enough time to adapt to the new potential of ${{\rm{H}}}_{2}^{+}$ and thus is prone to stay in the ground state of ${{\rm{H}}}_{2}^{+}$ after the interaction. However, in the intermediate regime (*γ* close to 1), there is no simple signature of electron-electron correlation: the populations in higher state of ${{\rm{H}}}_{2}^{+}$ depend strongly on laser frequency, intensity, internuclear distance and on the angle between the laser polarization and the molecular axis. In real molecules, the nuclear movement would affect the electron correlation during the tunneling ionization, which however should be small due to distinct movement time scales for nuclei and electrons.

## Data Availability

The datasets generated during and/or analyzed during the current study are available from the corresponding author on reasonable request.
